# Endoscopic Duodenal Stenting for Iatrogenic Stapfer Type I Duodenal Perforation: An Alternative to Surgical Repair

**DOI:** 10.7759/cureus.81612

**Published:** 2025-04-02

**Authors:** Ishraq Joarder, Shukria Ahmadi, Fahd Jowhari

**Affiliations:** 1 Faculty of Medicine, University of British Columbia, Vancouver, CAN; 2 Division of Gastroenterology, Kelowna General Hospital, Kelowna, CAN; 3 Southern Medical Program, University of British Columbia, Kelowna, CAN

**Keywords:** cre balloon dilation, duodenal perforation, ercp, stapfer type 1, stent

## Abstract

Endoscopic retrograde cholangiopancreatography (ERCP) is an essential therapeutic tool but carries risks of serious complications, including luminal perforation. Stapfer Type I perforations, involving lateral or medial duodenal wall tears, are traditionally managed surgically with high associated morbidity/mortality. Here, we report the first documented case to our knowledge of an iatrogenic post-dilation Stapfer Type I duodenal perforation successfully managed with placement of a fully covered self-expandable metallic stent (Fc-SEMS). An 87-year-old female with a history of chronic obstructive pulmonary disease, coronary artery disease, hypertension, and hypothyroidism presented with cholestatic transaminitis and suspected biliary obstruction. Endoscopic evaluation during ERCP revealed a pre-ampullary peptic duodenal stricture not traversable with the duodenoscope. Endoscopic dilation with a CRE balloon led to a duodenal perforation and a through-the-scope duodenal Fc-SEMS was deployed bridging the perforation. The patient demonstrated rapid clinical and radiologic improvement, with full recovery and successful stent removal in two months. This case highlights Fc-SEMS as a viable, less invasive alternative to surgery for high-risk patients who have sustained a duodenal perforation.

## Introduction

Endoscopic retrograde cholangiopancreatography (ERCP) is a widely utilized procedure for both diagnostic and therapeutic management of pancreaticobiliary pathologies, including choledocholithiasis, strictures, and malignancies. A recent systematic review and meta-analysis published in 2025 of ERCP-related adverse events reported an overall mortality rate of 0.2% [1]. Other adverse events, in descending order of incidence, included pancreatitis (4.6%), cholangitis (2.5%), bleeding (1.5%), cholecystitis (0.8%), and perforation (0.5%) [1]. Interventions performed during ERCP, such as balloon dilations, have an overall incidence of perforation of about 1.6% [2]; however, in patients with specific risk factors such as esophageal or gastric strictures, this can reach up to 16.7% [2,3]. While remaining relatively uncommon, ERCP-related perforations are associated with mortality rates up to 20% [4,5].

ERCP-related perforations are commonly classified using the Stapfer system, which categorizes injuries based on their anatomical location and mechanism. Type I perforations, characterized by injuries to the lateral or medial duodenal wall, typically from endoscopic maneuvers, are traditionally managed surgically [6-8]. Type II periampullary perforations are commonly caused by sphincterotomies, and type III pancreatic/bile duct perforations are typically caused by wire/basket instrumentation [6,7]. The management of both types II and III injuries is widely debated, with a growing shift towards non-surgical and endoscopic approaches, such as fully covered self-expandable metallic stent (Fc-SEMS), though surgical intervention remains necessary in select cases [5-9]. Type IV perforations, often caused by excessive insufflation, require retroperitoneal air and are typically managed medically [5-7].

We present a case of an 87-year-old female who sustained an iatrogenic Stapfer type I duodenal perforation following stricture dilation during an attempted ERCP for suspected biliary obstruction. To our knowledge, this appears to be the first reported case of a Stapfer type I duodenal perforation successfully managed using Fc-SEMS. This case report highlights the potential role of Fc-SEMS as a viable endoscopic alternative to surgical intervention for type I duodenal perforations, particularly in frail, high-risk patients who may not be ideal candidates for surgery.

## Case presentation

An 87-year-old female, residing in long-term care, presented with a two to three week history of weakness, lethargy, upper abdominal pain, and acute on chronic dyspnea. Her medical history was notable for chronic obstructive pulmonary disease (COPD) requiring chronic home oxygen therapy (baseline 2L), coronary artery disease with a prior non-ST-elevation myocardial infarction (NSTEMI), hypertension, hypothyroidism, dementia, a right hip fracture status - post open reduction and internal fixation (ORIF), a duodenal ulcer diagnosed in her 30s, gastroesophageal reflux disease (GERD)-treated intermittently with ranitidine, and a previous upper gastrointestinal bleed related to peptic ulcer disease.

Upon admission, the patient was afebrile, exhibited localized right upper quadrant tenderness, and her lab examinations demonstrated mixed, but predominantly cholestatic transaminitis (Table 1). She also had mild leukocytosis and an elevated C-reactive protein (CRP), but was otherwise vitally stable (Table 1). Empiric antibiotic treatment with renally dosed piperacillin-tazobactam 2.25g IV q6h was initiated.

**Table 1:** Relevant laboratory investigations upon admission WBC: white blood count; CRP: C-reactive protein; ALT: alanine aminotransferase; AST: aspartate aminotransferase; GGT: gamma-glutamyl transferase; ALP: alkaline phosphatase

Investigation	Patient's Result	Reference Range
WBC (x10^9^/L)	14.4	4-10
CRP (mg/L)	151.2	<10
Bilirubin Total (μmol/L)	50	<18
ALT (U/L)	203	<52
AST (U/L)	163	<40
GGT (U/L)	351	<36
ALP (U/L)	416	34-104

Abdominal ultrasound revealed common bile duct dilation up to 9 mm with some new extrahepatic biliary tree thickening, suspicious for cholangitis. The gallbladder had some thickening with debris as well. The spleen and liver were normal. A previous ultrasound completed two years prior showed low-grade acute cholecystitis and choledocholithiasis, which was not producing significant obstruction and was managed conservatively.

**Figure 1:**
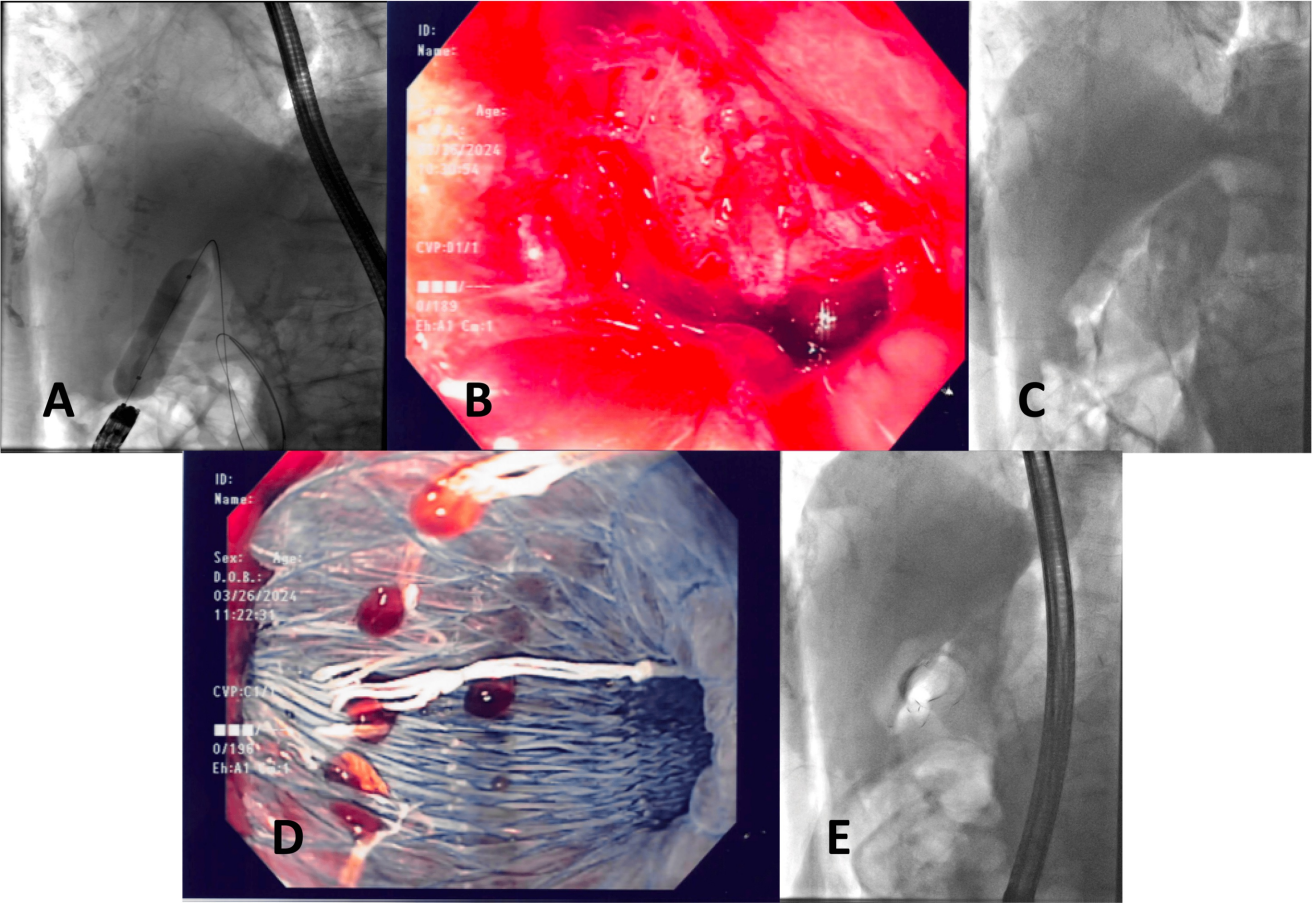
Fluoroscopic and endoscopic images A: Endoscopic balloon dilation of the peptic duodenal stricture using a controlled radial expansion (CRE) balloon. B: Endoscopic visualization of the luminal perforation in view (lumen positioned at 12 o’clock). C: Radiographic evidence of extraluminal air surrounding the liver, indicative of duodenal perforation. D: Deployment of a fully covered duodenal stent across the stricture. E: Fluoroscopic confirmation of the duodenal stent in optimal position.

With the suspicion of possible choledocholithiasis, an ERCP was arranged. Intraprocedurally, a peptic stricture from associated duodenal ulcerations in the D1/2 junction was found. As the ampulla was inaccessible, the stricture was dilated using a controlled radial expansion (CRE) balloon to provide access and complete the procedure. Under fluoroscopic and endoscopic guidance, a wire was advanced into the third and fourth portions of the duodenum, and the stricture was dilated in sequential increments up to 18 mm (Figure 1A). Upon dilation, there was immediate endoscopic/fluoroscopic evidence of a duodenal perforation of unclear thickness with free air seen around the liver (Figures 1B, 1C). A 20 mm x 60 mm fully covered metal Niti-S duodenal stent was placed across the perforation (proximal extent in the duodenal bulb), and a 16-FR nasogastric tube was inserted into the antrum of the stomach for decompression (Figures 1D, 1E). Post procedure, the patient was transferred to recovery with stable hemodynamics. Her abdomen was soft and non-tender, and she was started on a proton pump inhibitor infusion.

**Figure 2:**
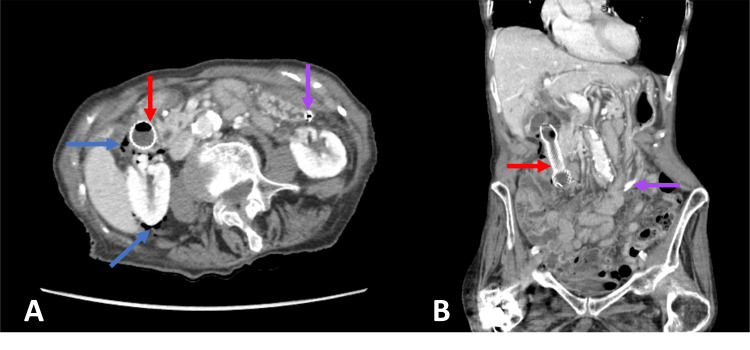
Abdominal computed tomography A and B: Axial and coronal CT abdomen pelvis with oral contrast demonstrating duodenal stent spanning the first and second parts of the duodenum (red arrow) and free air surrounding the right retroperitoneum and pararenal regions (blue arrow). Nasogastric tube tip in the gastric antrum (purple arrow) with a collapsed stomach seen proximally.

CT abdomen pelvis demonstrated diffuse retroperitoneal free air and focal thinning of the postero-lateral duodenal wall at the site of perforation (Figure 2). The common bile duct was not significantly dilated, and the stent was seen in good position. It was determined that close monitoring, rather than surgical intervention, was indicated as the endoscopically placed duodenal stent was effectively covering the perforation.

**Figure 3:**
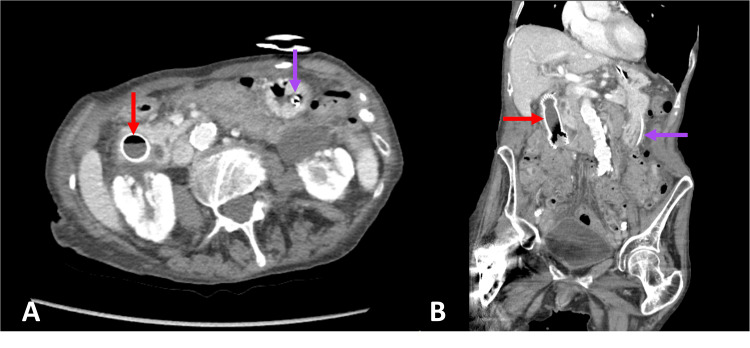
Abdominal computed tomography A and B: Axial and Coronal CT Abdomen Pelvis with contrast demonstrating almost complete resolution of retroperitoneal free air with only few residual locules remaining. Duodenal stent (red arrow) and nasogastric tube (purple arrow) have remained in their respective positions with no migration noted.

Two days later, repeat CT abdomen pelvis demonstrated near-complete resolution of retroperitoneal free air (Figure 3). Follow-up blood work also revealed normalization of cholestasis with normal bilirubin, transaminases, and only mild elevations in GGT and ALP, despite the stent traversing the papilla, suggesting that the biliary obstruction had spontaneously resolved and the stent was not occluding the ampulla. Her leukocytosis was also resolving, and her blood/urine cultures remained negative throughout her hospital course. Altogether, her initial presentation and subsequent resolution of symptoms following duodenal stricture dilation suggested that a combination of the duodenal stricture, ulceration, and resulting transient cholangitis was likely causing her initial obstructive symptoms.

**Figure 4:**
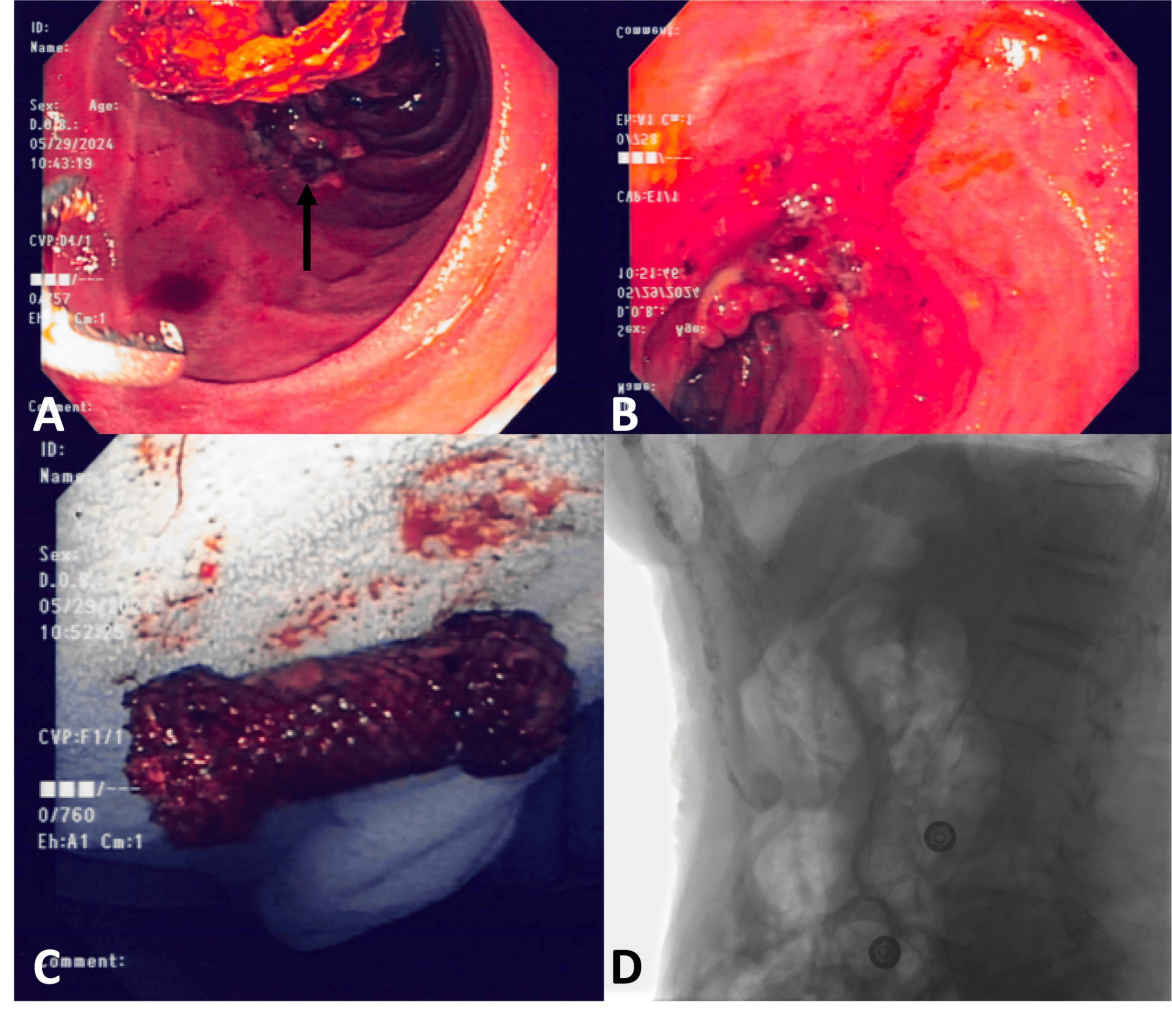
Endoscopic and radiographic images after perforation healed A: Collapsed duodenal stent seen above healed site of perforation (black arrow), with open forceps seen ready for removal. B: Endoscopic visualization of the healed perforation site with granulation tissue formation. C: Fully intact stent following successful retrieval. D: Radiographic imaging demonstrating the absence of free air, confirming resolution of perforation.

She was discharged in stable condition after a one-week hospital stay with a plan to remain on pantoprazole 40 mg BID until she returned in two months for removal of her duodenal stent. She did remarkably well in the interim and tolerated an oral diet with no evidence of duodenal/gastric outlet obstruction. The duodenal stent was successfully removed after eight weeks, and the perforation site had healed well (Figure 4). There were no signs of major ulcerations remaining, and she was instructed to continue her pantoprazole 40 mg BID dose lifelong.

## Discussion

Duodenal perforations are among the most serious ERCP-related complications, often associated with significant morbidity/mortality. The most recent American Gastroenterological Association (AGA) clinical practice guidelines reviewing endoscopic management of perforations in the gastrointestinal tract provide recommendations for both Stapfer type I and II duodenal perforations [10]. 

Currently, surgery remains the mainstay recommended treatment for large type I duodenal perforations (lateral duodenal wall tear > 3 cm), although endoscopic closure feasibility should still be assessed while awaiting urgent surgical consultation [10]. Despite surgical intervention, post-operative mortality rates for Stapfer type I perforations remain high, with a recent retrospective study reporting rates of 17% in 100 operatively managed cases [11]. Minimally invasive endoscopic techniques have had significant advancements recently with reports of successful endoscopic closure using through-the-scope (TTS) or over-the-scope (OTS) clips (for perforations < 2 cm), endoloops or suturing (for perforations > 2 cm), and fibrin glue [8,10-14]. These techniques have demonstrated >90% clinical success rates, precluding the need for surgical intervention in many cases [10,11,15]. However, some of these advanced techniques, such as suturing, may not be widely available.

Unlike type I duodenal perforations, the use of endoscopic techniques as first-line management for type II duodenal perforations is already well-established [10,16]. Among these techniques, the placement of Fc-SEMS to seal perforations has demonstrated high clinical success for type II injuries, avoiding the risks associated with surgery [9,10,17]. However, from our literature review, this technique has not previously been demonstrated in type I perforations. Our case highlights the first reported use of Fc-SEMS to successfully treat a Stapfer type I duodenal perforation, offering a promising alternative for select cases where surgical intervention poses high risks.

The decision to manage this case conservatively was influenced by several patient-specific factors. Firstly, the patient’s advanced age, frailty, and significant comorbidities, including cognitive impairment and chronic oxygen requirements, placed her at a very high risk for post-surgical complications. Furthermore, the perforation was identified and managed early during the procedure, with immediate placement of an Fc-SEMS to contain the defect. This approach was reinforced by the rapid clinical improvement observed, including resolution of retroperitoneal free air and normalization of laboratory findings.

The placement of Fc-SEMS provides several advantages. The stent offers immediate mechanical sealing of the perforation, preventing further leakage of gastrointestinal contents and facilitating tissue healing [18,19]. Unlike surgical repair, this approach avoids risks associated with prolonged general anesthesia, postoperative infections, and delayed recovery times, making it advantageous in patients with significant comorbidities. Furthermore, the removability of Fc-SEMS enables a temporary intervention without permanent alteration to the gastrointestinal anatomy. 

Despite these benefits, the use of Fc-SEMS is not without risks. Stent migration is a well-documented complication, occurring in 20-40% of cases in some studies [20]. Migration can lead to inadequate sealing of perforations, requiring repeat endoscopic interventions or conversion to surgery. Other potential concerns include duodenal wall erosions, luminal obstruction, and difficulty in stent removal [20]. Careful patient monitoring and timely stent removal are essential to mitigate these risks. Another potential complication is ampullary obstruction from the stent. In our case, the patient’s transaminases continued to improve, suggesting that there was enough room in the periampullary area to allow for adequate biliary drainage and prevent cholestasis.

## Conclusions

Our case supports the growing role of endoscopic interventions in managing gastrointestinal perforations and highlights a unique case of endoscopic management of type I duodenal perforations. While surgical repair remains necessary for large/unstable perforations, endoscopic techniques can offer a viable alternative for select patients. Further research is needed to establish standardized criteria for patient selection, optimization of stent placement techniques, and for the assessment of long-term outcomes of endoscopic management in type I duodenal perforations.
